# The United States must improve its data infrastructure to ensure high-quality mental health care

**DOI:** 10.3389/frhs.2023.1059049

**Published:** 2023-02-28

**Authors:** Tami L. Mark

**Affiliations:** RTI International, Durham, NC, United States

**Keywords:** psychiatric, treatment outcomes, follow-up studies, quality, health services, suicide

## Abstract

Use of and spending on mental health services in the United States more than doubled over the past two decades. In 2019, 19.2% of adults received mental health treatment (medications and/or counseling) at a cost of $135 billion. Yet, the United States has no data collection system to determine what proportion of the population benefited from treatment. Experts have for decades called for a learning behavioral health care system: a system that collects data on treatment services and outcomes to generate knowledge to improve practice. As the rates of suicide, depression, and drug overdoses in the United States continue to rise, the need for a learning health care system becomes even more pressing. In this paper, I suggest steps to move toward such a system. First, I describe the availability of data on mental health service use, mortality, symptoms, functioning, and quality of life. In the United States, the best sources of longitudinal information on mental health services received are Medicare, Medicaid, and private insurance claims and enrollment data. Federal and state agencies are starting to link these data to mortality information; however, these efforts need to be substantially expanded and include information on mental health symptoms, functioning, and quality of life. Finally, there must be greater efforts to make the data easier to access such as through standard data use agreements, online analytic tools, and data portals. Federal and state mental health policy leaders should be at the forefront of efforts to create a learning mental health care system.

## Introduction

Over the past 50 years, behavioral health policymakers and advocates in the United States have focused on expanding insurance coverage of mental treatment, which has traditionally been limited relative to coverage of other diseases (1, 2). For example, the Mental Health Parity and Addiction Equity Act of 2008 (MHPAEA) required that health plans cover behavioral health services no less generously than other conditions. Additionally, mental and substance use disorder services were included as an “essential benefit” under the Affordable Care Act enacted in March 2010. Because of these efforts, the share of individuals without mental health insurance is at historic lows (3).

Expanded insurance coverage, and the development of new mental health therapies such as antidepressants, spurred increased use of and spending on mental health treatment. The percentage of adults receiving mental health treatment increased from 12.6% in 2004 to 19.2% in 2019 (3, 4). Spending on mental health treatment more than tripled (from $41 billion to $135 billion) from 2000 through 2019 (5, 6).

Yet, while more individuals are receiving treatment than ever before, mental health-related morbidity and mortality is rising, rather than diminishing. For example, from 1999 to 2020, the suicide rate increased from 10.5 per 100,000 to 13.5 per 100,000, substantially above the Healthy People 2020 target of 10.2 per 100,000 (7, 8). The prevalence of depression is also growing (9–11), and drug overdose deaths continue to rise at alarming rates: from 6.1 per 100,000 in 1999 to 28.3 in 2020 (12, 13). Prior to the COVID-19 pandemic, suicide and overdoses were the main reason why life expectancy in the United States fell for three consecutive years, from 78.9 years in 2014, to 78.6 in 2017, after rising for decades (14).

Many argue that the solution to reducing suicides and overdoses is to deliver more treatment. However, behavioral health workforce shortages and rising health care costs make expanding treatment challenging. Moreover, it is well documented that the quality of mental and substance use disorder treatment in the United States is often sub-par. To identify solutions to achieving better mental health treatment outcomes with limited resources, experts have for decades called for a learning behavioral health care system: a data collection system that generates knowledge and applies it to improve clinical and public health practice (15, 16).

In this paper, I suggest steps to improve the United States data infrastructure to create a learning system. I propose that such a system should be characterized by longitudinal data that allow analyses of the association between treatment received and treatment outcomes (i.e., death, symptoms, functioning, and quality of life). First, I describe the types of data needed to create a learning health care system. I highlight that mortality data are already available, while other types of outcomes need to be routinely collected through surveys or registries or pulled from electronic medical records. Next, I describe how mortality data and other outcomes could be linked to insurance claims data. I conclude by discussing the need for making data easier to access, such as by developing and promoting standard data use agreements, analytic tools, and data portals.

## Data elements needed and potential data sources

Table 1 describes the data elements needed to ensure an accountable mental health care system that delivers high-quality services. The first required type of data elements capture what *mental health care services* individuals are receiving over time. Outside of the Veterans Administration, the most robust source of longitudinal data on treatment services are insurance claims and enrollment records. These capture the largest populations and the most detail on types and dates of health care services received using well-established taxonomies, such as Current Procedure Terminology (CPT) Codes and National Drug Codes (NDCs). Insurance claims are segmented by payer type: Medicare, Medicaid, and private claims. Medicaid covers about 18% of individuals in the United States, Medicaid another 18%, and private health insurance 66% (17). The Federal government can most readily access Medicare and Medicaid insurance data because Medicare and Medicaid are Federal and Federal-state programs, respectively.

**Table 1:** Data elements and potential data sources to create a learning mental health care system.

Data Element Type	Potential Data Source
Health care services received	Medicare, Medicaid, private insurance claims data
Mortality	Vital statistics
Symptoms, functioning, quality of life	Government-mandated registries
Symptoms, functioning, quality of life	Electronic medical records
Symptoms, functioning, quality of life, experience of care, activities that may improve outcomes (e.g., mindfulness, exercise, diet, social engagement)	Surveys
Environment (e.g., access to healthy food, clean air, safe neighborhoods, employment, financial stability)	Census data and surveys

The next required data element is *mortality* information. Because death rates from mental health and substance use disorders are important, common, and may be preventable with treatment, they should be a key focus of a learning health care system. In 2019, there were 75,00 overdose-related deaths, 47,000 suicide-related deaths, and 39,000 alcohol-related death (18). In the United States, death data is collected by designated professionals (e.g., physicians, coroners, forensic pathologists) using standardized death certificates that capture cause of death, demographics, and social security number (19). Death certificates are transmitted to state public health departments as part of their vital statistics data collection efforts and then sent onto a central repository at the Federal Centers for Disease Control and Prevention (20).

Data on other outcomes, such as mental health symptoms, functioning, and quality of life can be collected through surveys or government-mandated registries, or can be extracted from electronic medical records. For example, Federal agencies such as the Substance Abuse and Mental Health Services Administration (SAMHSA) and the Centers for Disease Control and Prevention (CDC) conduct annual national- and state-level surveys that capture information on *mental health conditions, symptoms, functioning, and quality of life*. An example of a government-mandated registry is the SAMHSA National Outcome Measures or NOMS registry. As a requirement of the SAMHSA Prevention and Treatment Block Grant (SABG), behavioral health providers that receive any public funding must report on a core set of outcome measures, including information on symptoms and functioning, at admission and discharge.

Another potential source for *outcomes* data are electronic medical records that are centrally collected in health information exchanges. For example, Maryland has a health information exchange called CRISP that pulls data from electronic medical records into a data repository (21). Increasingly, behavioral health providers are collecting patient-reported outcome measures such as depression scores using the PHQ-9 and anxiety symptoms using the GAD-7. These data could be pulled into the health information exchanges, linked to service data, and analyzed to understand the relationship between services received and outcomes.

A robust learning mental health care system would not only provide data to understand the relationship between the receipt of mental health services and outcomes, but would also illuminate how *personal behaviors* and *environmental conditions* affect outcomes. For example, exercise, mindfulness training, social connectedness, and diet are behaviors that individuals may pursue outside of the formal mental health system that could improve their mood and other mental health symptoms (22–25). Information on these behaviors could come from Federal surveys, such as the National Survey on Drug Use and Health and the Behavioral Risk Factor Surveillance Survey. Furthermore, environmental factors such as access to healthy food, clean air, safe neighborhoods, employment, and financial stability may influence mental health outcomes. Some of these data elements on neighborhood characteristics are collected by the Census Bureau's American Community Survey.

## Linking mortality data to longitudinal treatment data

Although drug overdose and suicide mortality rates have been closely tracked by Federal and state governments, they are not routinely linked to longitudinal health care service use data to facilitate analyses of the relationship between treatment received and mortality outcomes (26, 27). Rather, research on the relationship between therapies received and mortality consist of ad-hoc, one-time studies typically using a single state's linked data. For example, studies using a single state's data have examined the relationship between receipt of opioid use disorder medications and all-cause and opioid-related mortality and the relationship between tapering opioid pain medications and rates of overdose and suicide (28–31). These one-time studies require months of time navigating and negotiating labyrinthian policies and procedures within state and Federal agencies. Even behavioral health departments within the same state must negotiate with their public health department to link mortality information to their treatment data sets.

The only health care system in the United States that routinely links outcome information to service delivery is the Veterans Health Administration. Published research demonstrates the power of these data. For example, McCarthy and colleagues found that implementation of a suicide risk identification algorithm was associated with greater treatment engagement and fewer mental health admissions, emergency department visits, and suicide attempts, but was not associated with less suicide or all-cause mortality. Similarly, Shiner and colleagues found no correlation between overall quality of outpatient mental health care and suicide death, concluding that other interventions may be necessary to reduce suicides. These results may not be generalizable to the greater United States population and cannot easily be replicated among non-VA populations because of the lack of longitudinal data linked to mortality outcomes.

In September 2022, the Federal government announced that it was making Medicaid insurance claims data linked to cause of death information available for the first time (32). The Federal government should support states to conduct similar linkages of their Medicaid data to their mortality information in the form of technical assistance, workforce assistance, and financing. Many, if not most, Departments of Behavioral Health have no easy access to Medicaid claims data that would allow them to identify opportunities to improve the quality and continuity of mental and addiction treatment. States should consider re-organizing their data governance approaches to streamline greater access and allow greater ongoing data and analytic coordination between state Departments of Behavioral Health and Medicaid Agencies.

While the linkage of Medicaid and Medicare data to mortality data would be a significant improvement, this still would leave the behavioral health system unable to answer basic questions about the 66% of Americans with private insurance (33). Private health insurance is the largest payer for behavioral health treatment, followed by Medicaid (34). A host of vendors license private sector data that they aggregate from private health plans (e.g., Fair Health, MarketScan, Health Cost Institute); however, these data are not linked to mortality information because of Center for Disease Control and Prevention's (CDC) and privacy concerns and resource constraints.

## Linking symptom and functioning data to longitudinal treatment data

Improving the mental health system's ability to reduce morbidity is equally as important as improving its ability to prevent mental health-related mortality. This will require linking data on symptoms, functioning, and quality of life to information that captures mental health services use, such as insurance claims data. One example of the benefits of such as a system comes from the English Improving Access to Psychological Therapies (IAPT) program, which delivers psychological therapies recommended by the National Institute for Health and Care Excellence for depression and anxiety disorders to more than 500,000 patients in the UK each year. A session-by-session outcome monitoring system obtains symptom scores before and after treatment for 98% of patients. Researchers have been able to use these data to identify factors associated with better depression and anxiety outcomes, including number of treatment sessions and shorter wait time to start treatment. These and other organizational factors accounted for 33% of variance in reliable improvement and 22% for reliable recovery.

CDC's National Center for Health Statistics recently established a record linkage program to “maximize the value of the Center's population-based surveys.” Through this program, they have recently linked the National Health Interview Survey (NHIS) and the National Health and Nutrition Examination Survey (NHANES) to Medicaid and Medicare claims data. The NHIS includes a battery of questions to assess mental health and anxiety symptoms. The NHANES includes information the 9-item Patient Health Questionnaire Depression screener. These data may be useful to begin to answer questions such as what percentage of people who received depression treatment had a resolution of their depressive symptoms.

## Facilitating the use of linked data for quality improvement

Creating data sets with information on service use linked to subsequent information on symptoms, functioning, quality of life, and mortality in and of itself will, of course, not improve the quality of mental health care in the United States. The data must be analyzed by Federal and state behavioral health agencies, health plans, and researchers to identify opportunities for quality improvement. This will require streamlining data governance and sharing processes and policies such as by using standard data sharing templates, allowing ongoing access rather than one-time access, allowing researchers and other to access data through a central portal (see, for example, ResDAC.org), allowing access through an Application Programming Interface (API), and providing tools to analyze and visualize the data without transferring it (see, for example, data.cms.gov and data.cdc.gov) (35–37).

## Caveats and limitations

One concern with linking digital data to capture more detail on individuals' mental health care service use and outcomes is that it offers more opportunity for privacy violations. Scientific advances in privacy preserving linkage techniques, such as block chain and encryption, may help to reduce the risk of disclosure of personal information (38). Another caveat with this approach is that it ignores the fact that laws and regulations regarding digital data ownership in the United States are in flux. For example, does the patient own their electronic health care data or does the provider? Does the patient own their private insurance data or does the employer or health plan? Creating a robust learning health care system may also require clarification on what authority the Federal government has over the type and frequency of data that states, health plans, and providers collect and whether it must be shared with the Federal government. Finally, creating knowledge is not a guarantee that it will be used to improve mental health services and outcomes. The gap between scientific knowledge and clinical practice has been well documented. Performance measurement systems that are designed to motivate and hold accountable clinicians and providers by showing them their performance and outcomes relative to their peers generally have small to moderate effects on healthcare quality, often because the recipients of the information do not know how to improve performance.

## Conclusions

This paper is primarily meant to be a call to action for Federal and state mental health policy leadership. Researchers, patients, providers, and others concerned with the quality of the United States mental health system can emphasize the need for a learning behavioral health care system to identify how to organize and deliver mental health services to obtain the best outcomes. One recent positive development is that Federal bodies are increasingly endorsing the need to link existing data on service use and outcomes. For example, in its 2017 report to Congress, the Interdepartmental Serious Mental Illness Coordinating Committee's recommended that public and private health care systems routinely link data on individuals with severe mental illness to mortality data (39). More broadly, CDC's data strategy emphasizes the need to increase data linkages across diverse data assets to facilitate public health surveillance and conduct longitudinal follow-up of individuals and cohorts of individuals (40). Leaders in Federal mental health agencies could piggyback on these broader health system initiatives.

## Data Availability

The original contributions presented in the study are included in the article/Supplementary Material, further inquiries can be directed to the corresponding author.
